# Phytochemical Content, Antibacterial Activity, and Antioxidant, Anti-Inflammatory, and Cytotoxic Effects of Traditional Medicinal Plants against Respiratory Tract Bacterial Pathogens

**DOI:** 10.1155/2023/1243438

**Published:** 2023-05-31

**Authors:** Mashilo M. Matotoka, Gabriel T. Mashabela, Peter Masoko

**Affiliations:** ^1^Faculty of Science and Agriculture, Department of Biochemistry, Microbiology and Biotechnology, University of Limpopo, Private Bag X1106, Sovenga 0727, South Africa; ^2^Faculty of Medicine and Health Sciences, Division of Molecular Biology and Human Genetics, Department of Biomedical Sciences, Stellenbosch University, P.O. Box 19063, Francie van Zijl Drive, Tygerberg 7505, South Africa

## Abstract

Respiratory tract infections (RTIs) are frequent ailments among humans and are a high burden on public health. This study aimed to determine the *in vitro* antibacterial, anti-inflammatory, and cytotoxic effects of indigenous medicinal plants used in the treatment of RTIs, namely, *Senna petersiana*, *Gardenia volkensii*, *Acacia senegal*, and *Clerodendrum glabrum*. Dried leaves were extracted using various organic solvents. Antibacterial activity was quantified using the microbroth dilution assay. Protein denaturation assays were used to evaluate anti-inflammatory activity. The cytotoxicity of the extracts towards THP-1 macrophages was evaluated using the 3-(4,5-dimethylthiazol-2-yl)-2,5-diphenyltetrazolium bromide (MTT) assay. Antioxidant activity was determined using free radical scavenging activity and ferric-reducing power. Total polyphenolics were quantified. Liquid chromatography mass spectrometry was used to evaluate the acetone plant extracts. Nonpolar extracts had noteworthy antibacterial activity against *Staphylococcus aureus*, *Escherichia coli*, *Pseudomonas aeruginosa*, and *Mycobacterium smegmatis* where MIC values ranged between 0.16 and 0.63 mg/mL. At 100 *μ*g/mL, *A. senegal*, *G. volkensii*, and *S. petersiana* had a nonsignificant effect on the viability of the THP-1 macrophages. The LC-MS analysis of the leaf extracts of *S. petersiana* detected *Columnidin,* Hercynine, L-Lysine citrate, and Gamma-Linolenate. A pentacyclic triterpenoid, cochalate, was detected in *G. volkensii.* Two flavonoids 7-hydroxy-2-(4-methoxyphenyl)-4-oxo-chroman-5-olate and (3R)-3-(2,4-dimethoxyphenyl)-7-hydroxy-4-oxo-chroman-5-olate were detected in the *C. glabrum* extract. The findings from this study indicated that the leaves of the selected plant extracts possess antioxidant, anti-inflammatory, and antibacterial activity. Therefore, they may serve as good candidates for further pharmaceutical investigations.

## 1. Introduction

Respiratory tract infections (RTIs) are frequent ailments among humans and are a high burden on public health. The effects of RTIs are most prevalent in developing countries in sub-Saharan Africa, where higher economic losses, morbidity, hospital admissions, and mortality rates are experienced [1]. Respiratory tract infections can be categorized into upper and lower tract infections and may be caused by bacteria, viruses, fungi, or parasites [1]. The majority of RTIs are transmitted mainly through inhalation of infected aerosols or contact with bodily fluids [1]. Lower respiratory tract infections (LRTIs) were counted among the top five causes of death in children younger than 5 years [1, 2]. Various opportunistic bacteria, including *Streptococcus pneumoniae, Haemophilus influenzae*, *Klebsiella pneumoniae*, *Staphylococcus aureus, Acinetobacter species*, *Streptococcus viridans*, *Pseudomonas aeruginosa*, *Escherichia coli,* and *Proteus* species have been identified as common causes of LRTIs [1–3].

Microbial infections have been associated with the upregulation of proinflammatory signal molecules and the release of lysosomal enzymes which cause tissue damage during prolonged inflammation [4]. Free radicals such as reactive oxygen species (ROS) contribute to the development of inflammatory diseases. Chemical species known as antioxidants are capable of scavenging ROS and have been linked to the remediation of inflammatory disorders [5]. Synthetic drugs such as rifampicin, erythromycin, benzylpenicillin, chloramphenicol, and tetracycline are used for the treatment of respiratory bacterial pathogens [6]. The drawback of conventional antibiotics is their associated side effects which include nausea, vomiting, diarrhoea, abdominal pain, loss of appetite, bloating, dizziness, and loss of appetite [7].

Medicinal plants have been the cornerstone of herbal medicine, whereby infusions, macerations, tinctures, and decoctions of different plant parts have been used to treat diseases [8]. The success of medicinal plants as an alternative source of therapy for humans stems from their ability to synthesize bioactive phytochemicals such as phlobatannins, anthocyanin, betacynin, flavonoids, tannins, phenolics, alkaloids, glycosides flavonoids, steroids, saponins, and terpenoids [9]. Phytochemicals play critical protective roles against pathogenic infections commonly caused by bacteria, fungi, viruses, parasites, herbivores, insects, pests, and harsh environmental conditions such as unfavourable climate and insufficient nutrient supply [10].

Bioactive phytochemicals in medicinal plant extracts have various structural complexities and may exert their therapeutic effects by acting on new targets [11]. Moreover, the additive and synergistic effects associated with phytochemicals may further provide a multitargeted therapeutic approach that might be more advantageous than single-target-based drugs [12]. Herbal medicine, a prominent form of traditional medicine, serves as a relief for populations in developing countries where health facilities such as hospitals, dispensaries, pharmacies, and clinics are either underdeveloped or expensive for patients [13–15].

Participatory collaboration with indigenous cultures plays a pivotal role in retrieving anecdotal information, identification, and documentation of medicinal plants used to treat various diseases [16]. Sub-Saharan Africa boasts an estimated 45, 000 plant species [17], of which 45.5% (20 456) are indigenous plant taxa spread across the Republic of South Africa. Only 10% (≈2062 plant species) of the total flora of South Africa has been recorded to be used in traditional medicine [18]. It has been reported that about 27 million of the South African population depend on traditional medicine [19].

*Gardenia volkensii* belongs to the Rubiaceae family and has common names such as bushveld gardenia, sand veld gardenia, savanna gardenia, and Transvaal gardenia [20]. This plant has hairless obovate leaves that are grouped at the tips of the short lateral twigs. The flowers are axillary, solitary, large, and showy in white. *G. volkensii* grows in warm temperate regions which include Africa, Madagascar, East and Southeast Asia, the western Pacific, and Hawaiian islands [21]. *G. volkensii* has a wide distribution in Africa in countries such as Angola, Botswana, Eswatini, Ethiopia, Kenya, Malawi, Mozambique, Namibia, Tanzania, Uganda, Somalia, South Africa, Zambia, and Zimbabwe. These countries represent 71.4% of countries in which *G. volkensii* is indigenous [20]. The fruits and roots of *G. volkensii* are widely used in ethnomedicine for the treatment of asthma, infertility, earache, sore eyes, epilepsy, headache [22], asthma, chest complaints, colds, pneumonia, sore throat, and tuberculosis [20]. The nutritional value of the fruits of *G. volkensii* is indicated by their consumption by various animals such as elephants, kudu, velvet monkeys, and baboons [22]. *Senna petersiana* belongs to the Fabaceae family and can grow to a height of 7 m with dark green leaves which have several leaflets facing opposite. The flowers are large and yellow, while the pods produced are green and flattened. Seventy-four percent (74%) of *Senna* species are largely distributed in America; Australia has about 13%; and Africa together with Madagascar constitutes 10% of this genus distribution [23]. *S. petersiana* leaves are traditionally used to treat malaria and typhoid fever, and the roots are used for the treatment of coughs, colds, syphilis, and helminthic infections [24]. The pods produced by *S. petersiana* have been reported to be consumed either fresh or cooked [24]. *Acacia senegal* belongs to the Fabaceae family and is a thorny shrub tree of 2–6 or even 12 meters high with very branched and ascending branches. Its leaves are green-grey, alternating, and bipinnate. The cream-coloured small flowers produce pods that are straight and slightly curved [25]. *A. senegal* is a drought-resistant species that commonly occurs in arid, semi-arid, and subtropical regions in sub-Saharan Africa such as Senegal, Cameroon, and Sudan [26, 27]. The distribution of *A. senegal* extends to the Arabian Peninsula, Pakistan, and India [28]. *A. senegal* is famous for its gum arabic, a dried exudate rich in soluble fibres, which emerges from slits made in the bark of the stems and branches [28]. The different parts of *A. senegal* can be used in the form of decoctions, macerations, or administered dry. This species is traditionally used for the treatment of colds, coughs, diarrhoea, dysentery, expectorant, gonorrhoea, sore throat, and disorders of the urinary tract [25, 27]. *Carissa bispinosa* is an evergreen densely bushed small tree with a height of up to 1–5 meters that belongs to the Apocynaceae family. The branched stem of this species is green in colour, spiny, and rich in white latex. The leaves are ovate/rounded and are positioned in an opposite-decussate arrangement. It produces small white/tinged pink fragrant flowers, and the fully ripened fruits are red in colour and ovoid in shape. Species of the Carissa genus are native to Africa, Indo-China, Australia, and New Caledonia [29]. The roots of *C. bispinosa* are extracted and drunk as cough and diarrhoea medicine [30]. Local people in KwaZulu natal, South Africa, depend on the sale of fruits from Carissa spp. These fruits would usually be sold in the summer months. The popularity of fruits is attributed to their nutritional content such as vitamin C, calcium, magnesium, and phosphorus [31]. Although the antioxidant activity of flavonoids from the fruit of *C. bispinosa* has previously been reported [32], there is little scientific documentation of the biological activities of the leaves of this plant. *Clerodendrum glabrum* belongs to the Lamiaceae family. Its common names include glory bower, bag flower, and bleeding heart [33]. The roots and the bark are usually dried, pulverised, and then boiled to treat ailments such as oral ulcers, diarrhoea, and coughs [34]. *C. glabrum* is widely distributed in Asia, Australia, America, and Africa [35].

There are a few to no reports of the biological activities of the plant species selected for this study, despite their wide traditional use. The focus of this study was to select under-explored traditionally used medicinal plants which may hold potential for the development of efficacious antibacterial RTIs caused by common pathogens such as *Staphylococcus aureus*, *Escherichia coli*, *Pseudomonas aeruginosa*, and *Mycobacterium tuberculosis*. *Staphylococcus aureus* causes bacteremia, endocarditis, skin and soft tissue infections, osteoarticular infections, joint infections, and pleuropulmonary infections [36]. *Pseudomonas aeruginosa* has been reported in bacteremia, ventilator-associated pneumonia, urinary and respiratory tract infections, and skin and soft tissue infections [37]. *E. coli* is the most frequent cause of bloodstream and urinary tract infections among Gram-negative bacteria [38]. We determined the antibacterial, antioxidant, anti-inflammatory, and cytotoxic effects of the selected medicinal plants and showed the potential the active extracts have for further studies involving the treatment of bacterial RTIs and their accompanying symptoms.

## 2. Materials and Methods

### 2.1. Chemicals and Reagents

The chemicals and reagents used were as follows: N-hexane (Merck, Cas#: 110-54-3), dichloromethane (Merck, Cas#: 75-09-2), acetone (Merck, Cas#: 67-64-1), chloroform (CHCl_3_) (Merck, Cas#: 67-66-3), and methanol (Merck, Cas#: 67-56-1), middlebrook 7H9 (Merck, Cas#: 49767) or 7H10 medium (Fluka M0178), glycerol (Fluka, Cas#: 49769), and oleic albumin dextrose catalase (OADC) growth supplement (Fluka, Cas#: M0553), nutrient broth (ThermoFisher scientific, Cas#: CM0001B), nutrient agar (ThermoFischer, Cas#: CM0003B), p-iodonitrotetrazolium chloride (INT) (Sigma-Aldrich, Cas#: 146-68-9), 2,2-diphenyl-1-picrylhydrazyl (DPPH) (Sigma-Aldrich, Cas#: 1898-66-4), L-ascorbic acid (Merck, Cas#: 50-81-7), potassium ferricyanide (Merck, Cas#: 13746-66-2), trichloroacetic acid (Merck, Cas#: 76-03-9), ferric chloride (ThermoFisher scientific, Cas#: 21218), bovine serum albumin (BSA) (Merck, Cas#: 9048-46-8), sodium chloride (NaCl) (Merck, Cas#: 7647-14-5), hydrochloric acid (HCl) (Merck, Cas#: 7647-01-0), diclofenac sodium (Sigma-Aldrich, Cas#: 15307-79-6), Royal Park Medical Institute-1640 (RPMI-1640) medium (ThermoFisher scientific, Cas#: 21875158), fetal bovine serum (FBS) (ThermoFisher scientific, Cas#: 16140071), phorbol 12-myristate 13-acetate (PMA) (Merck, Cas#: 16561-29-8), dimethyl sulfoxide (DMSO) (Sigma-Aldrich, Cas#: 67-68-5), Dulbecco's Modified Eagle's Medium (DMEM) (ThermoFisher scientific, Cas#: 30030), 3 (4, 5 dimethylthiazol-2yl)-2-5-diphenyl tetrazolium bromide (MTT) (Sigma-Aldrich, Cas#: 298-93-1), Folin–Ciocalteu's reagent (Sigma-Aldrich, Cas#: 109001), sodium carbonate (Merck, Cas#: 497-19-8), gallic acid (Merck, Cas#: 149-91-7), sodium nitrite (NaNO_2_) (Merck, Cas#: 7632-00-0), aluminium chloride (AlCl_3_) (Merck, Cas#: 7784-13-6), quercetin (Merck, Cas#: 849061-97-8), sodium hydroxide (NaOH) (Merck, Cas#: 1310-73-2), bromocresol green (Merck, Cas#: 76-60-8), atropine (Sigma-Aldrich, Cas#: 51-55-8), sodium acetate (C_2_H_3_NaO_2_) (Merck, Cas#: 127-09-3), and vanillin (Merck, Cas#:121-33-5).

### 2.2. Plant Collection, Drying, and Storage

The selection of the plants in this study was based on their ethnopharmacological use for the treatment of respiratory infections and the management of accompanying symptoms.  Table 1 shows the selected plants and their traditional uses. The plants were collected at the Lowveld National Botanical garden located in Mbombela, Mpumalanga, South Africa. The identities of the plants were verified by botanist Dr. Bronwyn Egan from the Larry Leach herbarium at the University of Limpopo, and subsequently, voucher specimens were prepared and deposited in the herbarium. The leaves were dried at ambient temperature. Once dried, the plant material was ground to a fine powder using a commercial blender. The powdered material was stored in airtight glass bottles in closed cabinet compartments, away from sunlight.

### 2.3. Plant Extract Preparation

One gram of each of the ground plant materials from the selected plants was extracted with 10 mL of n-hexane, dichloromethane, acetone, and methanol in different 50 mL polyester centrifuge tubes. The tubes were shaken at 200 rpm in a shaking incubator for 30 mins. Decoctions of the plant leaves were prepared by boiling 1 g of ground leaves in 10 mL distilled water for 5 min. The extracts were filtered from the plant material into glass vials. The solvents were evaporated under a stream of air at ambient temperature. The extracts were quantified and reconstituted to a concentration of 10 mg/mL using acetone to prepare stock solutions.

### 2.4. Screening of Antimicrobial Activity

#### 2.4.1. Microorganisms and Media

Five microorganisms, namely *Staphylococcus aureus* ATCC 29213 and *Escherichia coli* ATCC 28922, *Pseudomonas aeruginosa* ATCC 27853, *Mycobacterium smegmatis* ATCC 1441, and *Mycobacterium tuberculosis* (H37Rv) were used. All the bacterial cultures were grown at 37°C. *M. smegmatis* and *M. tuberculosis* were cultured in Middlebrook 7H9 or 7H10 medium mixed with glycerol and an oleic albumin dextrose catalase (OADC) growth supplement for mycobacteria. All other microorganisms were cultured in nutrient broth/agar.

#### 2.4.2. Broth Microdilution Assay

The broth microdilution assay described by Eloff [40] was used. The antimicrobial activity of the plant extracts was evaluated using *E. coli* (2 × 10^10^ cfu/mL), *S. aureus* (2 × 10^8^ cfu/mL), *M. smegmatis* (2 × 10^5^ cfu/mL), *M. tuberculosis* (2 × 10^5^ cfu/mL), and *Pseudomonas aeruginosa* (3 × 10^9^ cfu/mL) as test microorganisms. The extracts were reconstituted to a concentration of 10 mg/mL using acetone. Sterile distilled water (100 *μ*L) was added to each well of a 96-well microtitre plate. The extracts (100 *μ*L) were serially diluted with distilled water in the 96-well microtitre plates to achieve a concentration of 0.02–2.5 mg/mL. Each microorganism culture (100 *μ*L) was separately added to each well aseptically. Rifampicin was used as a positive control for antimycobacterial activity, and tetracycline was used as a positive control for antibacterial activity. Sterile distilled water was used as the negative control. The microtitre plates were incubated for 24 h at 37°C for *E. coli*, *S. aureus*, *M. smegmatis*, and *P. aeruginosa*. After incubation, 40 *μ*L of 0.2 mg/mL of p-iodonitrotetrazolium chloride (INT) dissolved in sterile distilled water was added to each well and further incubated for 30 min. The plates inoculated with *M. tuberculosis* were incubated for 5 days after adding to the extracts and after incubation, 20 *μ*L of 0.02 mg/mL resazurin dissolved in distilled water was added. The plates were incubated for 4 h for optimal colour development. The minimum inhibitory concentrations (MIC) were determined as the lowest concentration of the plant extract that was able to inhibit bacterial growth. Total antibacterial activity was calculated as the total mass (mg) of the extract divided by the MIC value (mg/mL). Total activity (TA) is expressed as mL and represents the volume to which the extract (obtained from 1 g of plant material) can be diluted and still maintain effective antibacterial activity [41].

#### 2.4.3. Minimum Bactericidal Concentration Determination

The procedure described by Senhaji et al. [42] to determine the minimum bactericidal concentrations (MBC) of the extracts was performed with some modifications. Briefly, the microtitre plates used for determining MIC values were incubated for an additional 24 h to make a cumulative 48 h of incubation. The inability of the microorganisms to grow was indicated by the clear wells and 10 *μ*L of the samples were subcultured on nutrient agar and incubated for 24 h at 37°C. MBC was determined to be the lowest concentration that showed no bacterial growth on the nutrient agar plates.

### 2.5. Antioxidant Screening

#### 2.5.1. Free Radical (DPPH) Scavenging Assay

The free radical scavenging activity of the plant extracts was quantified using the 2,2-diphenyl-1-picrylhydrazyl (DPPH) method reported by Chigayo et al. [43]. Different concentrations of the extracts (15.63–250 *μ*g/mL) were prepared in a volume of 1 mL of solution. L-ascorbic acid was used as a positive control by preparing it in the same concentration range as the extracts. To these 1 mL solutions, 2 mL of 78.5 *μ*g/mL DPPH solution were added and vortexed thoroughly. All the prepared mixtures were left to stand in the dark for 30 min. The control solution was prepared by adding 2 mL of 78.5 *μ*g/mL DPPH to 1 mL of distilled water. After the elapsed time, the solutions were analysed with a UV/VIS spectrophotometer. The absorbance of the solutions was read at 517 nm.

(1) $$\begin{array}{c} \%scavenging\;activity=\frac{\left(Ac-As\right)}{Ac}\times 100\text{,} \end{array}$$

where Ac is the absorbance of the control solution and As is the absorbance of the extracts.

#### 2.5.2. Ferric-Reducing Power Assay

The ferric-reducing power of extracts was determined by using the procedure detailed by Vijayalakshmi and Ruckmani [44] which measures the formation of Pearl's Prussian blue at 700 nm. Varying concentrations of the extracts (39–625 *μ*g/mL) were prepared in 2.5 mL aliquots in test tubes and mixed with 2.5 mL sodium phosphate buffer (0.2 M, pH 6.6), followed by 2.5 mL of 1% aqueous potassium ferricyanide. The mixtures were vortexed after the addition of solutions. The mixtures were incubated at 50°C for 20 min. Two millilitres of 10% w/v aqueous trichloroacetic acid were added to the test tubes after incubation. The mixtures were centrifuged at 3000 rpm for 10 min, and 5 mL of the resulting supernatant was transferred to clean test tubes. To these solutions, 5 mL of distilled water and 1 mL of 0, 1% aqueous ferric chloride were added consecutively, with thorough mixing after each addition. A UV/VIS spectrophotometer was used to read the absorbance of solutions at 700 nm wavelength. The blank for this procedure was prepared in the same manner; however, the extracts were replaced by an equal amount of distilled water. L-ascorbic acid was used as a standard.

### 2.6. Anti-Inflammatory Activity Screening

#### 2.6.1. Bovine Serum Albumin Denaturation Inhibition Assay

The anti-inflammatory activities of the plant extracts were determined using a BSA assay reported by Bailey-Shaw et al. [45]. Bovine serum albumin (BSA) solution (0.5%, w/v) was prepared in 0.05 M tris-buffered saline. Tris-buffered saline was prepared by preparing 0.05 M Tris and 0.15 M sodium chloride, and the pH was adjusted to 6.4 with 0.5 M hydrochloric acid (HCl). Stock solutions of each plant extract were reconstituted in their appropriate solvent at a concentration of 10 mg/mL. Various concentrations of test solutions (0.25–2 mg/mL) of extracts were taken, respectively, in a volume of 50 *μ*L and mixed with 950 *μ*L (0.5% w/v BSA). The product (negative) control solution (1000 *μ*L) consisted of 950 *μ*L of 0.05 M tris-buffered saline and 50 *μ*L of each extract solution to consider the colouration of the extracts. BSA solution (0.5%) 950 *μ*L with 50 *μ*L of tris-buffered saline was used as the test solution control. The test solution control represented 100% protein denaturation. The results were compared with diclofenac sodium as a standard. The solutions were then heated in a heat block at 72°C for 5 min and cooled for 20 min under laboratory conditions. The turbidity of the solutions (level of protein precipitation) was measured at 660 nm in a UV/VIS spectrophotometer. Tris-buffered saline (0.05 M) was used as a blank. The experiments were conducted in triplicate, and the mean absorbance values were recorded. The percentage inhibition of precipitation (protein denaturation) was determined on a percentage basis.

(2) $$\begin{array}{c} \%inhibition=\frac{\left(Ac-As\right)}{Ac}\times 100\text{,} \end{array}$$

where Ac is the absorbance of the control solution and As is the absorbance of the extracts.

#### 2.6.2. Egg Albumin Denaturation Inhibition Assay

The reaction mixture (5 mL) consisted of 0.2 mL of egg albumin (from fresh hen's egg), 2.8 mL of 0.05 M tris-buffered saline (pH 6.4), and 2 mL of varying concentrations (0.25–2 mg/mL) of the plant extracts and standard drug (diclofenac sodium) (1, 0.5, 0.25 mg/mL). The product (negative) control solution (5 mL) consisted of 3 mL of 0.05 M Tris-buffered saline and 2 mL of each extract solution to consider the colour of the extracts when using the UV/Vis spectrophotometer. Egg albumin solution (0.2 mL and 4.8 mL tris-buffered saline) was used as the test solution (positive control). The positive control represented 100% protein denaturation. The mixtures were incubated at 37 ± 2°C in a biochemical oxygen demand (BOD) incubator for 15 min and then heated at 70°C for 5 min. After heating, the solutions were allowed to cool to room temperature for 30 min. After cooling, their absorbance was measured at 660 nm by using the tris-buffered saline as a blank [46]. The percentage inhibition of protein denaturation was calculated by using the following formula:

(3) $$\begin{array}{c} \%\;anti\;denaturation\;activity=\frac{\left(Ac-As\right)}{Ac}\times 100\text{,} \end{array}$$

where Ac is the absorbance of the control solution and As is the absorbance of the extracts.

### 2.7. Cell Viability Assay

The THP-1 cell line was cultured in Royal Park Medical Institute-1640 (RPMI-1640) medium, supplemented with 10% fetal bovine serum (FBS) and incubated in a 5% CO_2_/95% air fully humidified atmosphere at 37°C. THP-1 monocytes were differentiated into macrophages by exposure to phorbol 12-myristate 13-acetate (PMA) [47]. The differentiation of the THP-1 cell line with PMA treatment has been previously reported to be optimal when incubated for 2–5 days [48]. It is vital to arrest the differentiation of the cell line after 48 hr treatment with PMA to increase macrophage markers [47]. Growing THP-1 monocyte culture was diluted to 2 × 10^5^ cell/mL in a 50 mL vial and pretreated with 25 *μ*L of 100 *μ*g/mL PMA to yield a final concentration of 50 ng/*μ*L in the 50 mL vial, and the cells were seeded at 2 × 10^5^ cell/mL (per well) in flat bottom 96-well plates for 72 h in 5% CO_2_ at 37°C to induce maturation of the monocytes into macrophage-like adherent cells [49]. THP-1 cells per well were seeded in 96-well plates to a final volume of 100 *μ*L. After treatment with PMA, the spent media was removed, the cells were washed with prewarmed 1X PBS, and fresh media was added followed by 24 h incubation [44]. For maintenance of the Vero cell lines, a confluent Vero cell monolayer with a cell density of 4 × 10^4^ cells/well (100 *μ*L) was seeded in 96-well plates and incubated at 37°C in a 5% CO_2_ incubator for 24 h.

Extract concentrations were prepared separately; briefly, each extract was presolubilized in dimethyl sulfoxide (DMSO) to give a stock solution of 200 mg/mL. The extracts were diluted with complete RPMI 40 or DMEM media to give final concentrations of 1000, 500, and 100 *μ*g/mL. Actinomycin D was used as a positive control. For the THP-1 macrophages, the culture medium was replaced by a new complete RPMI medium containing plant extracts with the concentrations, and DMEM medium containing plant extracts with the concentrations was used for the Vero cell monolayer. The plates were incubated for another 24 h at 37°C in a 5% CO_2_ incubator, and the cell viability was evaluated using the MTT colourimetric assay. A 20 *μ*L of MTT solution 5 mg/mL in filter-sterilised phosphate-buffered saline (PBS) was pipetted into each well followed by a 4 h incubation period at 37°C in the 5% CO_2_ incubator until purple precipitates were visible under a microscope. The medium together with MTT was aspirated off the wells, and 100 *μ*L of DMSO was added to the wells to dissolve the formazan. The absorbance was determined at 570 nm using a microplate reader. The absorbance of wells filled with media alone was used as a blank. Results were obtained from three independent experiments, and duplicate assays were performed for each experiment [50].

### 2.8. Phytochemical Study of Plant Extracts

#### 2.8.1. Determination of Phenolic Content

The Folin–Ciocalteu reagent method described by Tambe and Bhambar [51] was adopted. Ten microliters of 10 mg/mL plant extracts were diluted with 490 *μ*L of distilled water, followed by the addition of 0.25 mL of Folin–Ciocaltleu reagent in each test tube. To this solution, 1.25 mL (7%) aqueous sodium carbonate (Na_2_CO_3_) was added, and the mixtures were incubated in the dark at ambient temperature for 30 min. The absorbance of the sample mixtures was measured at 725 nm. A blank was prepared similarly. Various concentrations of gallic acid (0.08–1.25 mg/mL) were prepared for the standard curve.

#### 2.8.2. Total Tannin Content Determination

To quantify the tannin content, the Folin–Ciocalteu method described by Tambe and Bhambar [51] was used. Briefly, 100 *μ*L of 10 mg/mL extracts were added to a clean test tube containing 7.5 mL of distilled water. The Folin–Ciocalteu reagent (0.5 mL) was added to the mixture and vortexed thoroughly. Ten millilitres (10 mL) of a 35% solution of sodium carbonate were added to the mixture. The mixture in the tube was transferred to a 10 mL volumetric flask, and the volume of the mixture was made up to 10 mL with distilled water. The mixture was shaken and kept at ambient temperature for 30 min in the dark. Gallic acid was used as a standard, and reference standard solutions (0.625–1 mg/mL) were prepared. The absorbance of the solutions was measured at 725 nm against a blank reagent blank. Tannin content was expressed as milligram gallic acid equivalence/Gram of extract (mg GAE/g). All the measurements were evaluated in triplicate.

#### 2.8.3. Total Flavonoid Content Determination

The method of Tambe and Bhambar [51] was used to quantify total flavonoid content. Briefly, 100 *μ*L of 10 mg/mL of plant extracts were added to 4.9 mL of distilled water in a clean test tube followed by the addition of 300 *μ*L of 5% sodium nitrite dissolved in distilled water. The mixture was allowed to react at ambient temperature for 5 min. Three hundred microliters (300 *μ*L) of 10% aluminium chloride (dissolved in distilled water) were added to the reaction mixture. The reaction was allowed to proceed for 5 min, after which 2 mL of 1 M sodium hydroxide was added. To construct a standard curve, a concentration gradient (16–125 *μ*g/mL) of quercetin was prepared, and each concentration was reacted in the same manner as the extracts. The absorbance of the samples was read against a reagent blank at a wavelength of 510 nm. The results are expressed in milligram quercetin equivalence/gram of extract (mg QE/g).

#### 2.8.4. Quantification of Total Alkaloid Content

A procedure detailed by Rao et al. [52] was followed. Working solutions of 1 mg/mL of each plant extract were prepared using dimethyl sulfoxide (DMSO). One milliliter (1 mL) of 2 M HCl was added to 1 mL of DMSO-dissolved extracts, and the resulting mixture was filtered using filter paper. The filtrate was transferred to a 250 mL separating funnel, and to this solution, 5 mL of 0.1% bromocresol green (dissolved in methanol) was added followed by 5 mL of phosphate buffer (pH 6.6). Chloroform (1 mL) was added into the separating funnel, and the mixture was vigorously shaken, after which the funnel was allowed to stand to allow the mixture to separate into different layers. The lower layer was collected in a 10 mL volumetric flask. The process was repeated with 2, 3, and 4 mL of chloroform. Atropine was used to construct a standard curve using a concentration range of 0.0625–1.0 mg/mL. The absorbance of the sample and standard solutions was recorded at a wavelength of 470 nm against a reagent blank. The total alkaloid content was expressed as milligram atropine equivalent/gram of extract (mg AE/g).

#### 2.8.5. Quantification of Total Flavonol Content

The aluminium chloride method detailed by Iqbal et al. [53] was followed to determine total flavonol content. Plant extracts were made to 1 mg/mL, and 0.5 mL of these solutions were added to test tubes followed by the addition of 0.5 mL of 2% aluminium chloride and 1.5 mL of 5% of sodium acetate. The solution was mixed well using a vortex. The solution was then transferred to a 2 mL Eppendorf tube and centrifuged for 20 min to obtain a clear solution. The absorbance of the solutions was recorded at a wavelength of 440 nm against a blank. Quercetin was used as a standard, and different concentrations (16–250 *μ*g/mL) were prepared. The results were expressed as mg quercetin equivalent per gram of extract (mg QE/g).

#### 2.8.6. Quantification of Total Proanthocyanidin Content

Total proanthocyanidin content was quantified by following the procedure described by Sun et al. [54]. In a test tube, 0.5 mL of 1 mg/mL of plant extracts were prepared. To this extract, 3 mL of 4% vanillin dissolved in methanol was added, followed by 1.5 mL of 37% HCl. The mixture was vortexed thoroughly and allowed to stand for 15 mins at ambient temperature. The absorbance of the solutions was measured at a wavelength of 500 nm against a reagent blank. Gallic acid was used as a standard, and concentrations of 16–250 *μ*g/mL were used to construct a standard curve. Total proanthocyanidin content was expressed as milligram gallic acid equivalence/gram of extract (mg GAE/g).

### 2.9. Liquid Chromatography-Mass Spectrometry (LC-MS) Studies

The LC-MS/MS analysis was carried out using a Waters Synapt G2 qTOF mass spectrometer. The Synapt G2 qTOF from Waters (Milford, USA) is a high-resolution quadrupole time-of-flight (qTOF) mass spectrometer capable of data independent analysis (DIA) using Waters ms E technology. When linked to a Waters Acquity UPLC, the system can achieve good chromatographic separation between compounds followed by simultaneous acquisition of both fragmented and unfragmented mass spectra of all compounds within each peak eluting off the column, together with UV spectra produced by the photodiode array (PDA) detector placed upstream of the qTOF. The acetone extracts were centrifuged at 12,000 rpm for 10 min before analysis. A waters HSS T3 column, 2.1 × 150 mm was used in obtaining the separation of the phytoconstituents. Two mobile phases (A) and (B) were used, where (A) consisted of 0.1% formic acid in water and (B) had acetonitrile 5 mM ammonium formate. A 5 *μ*l volume of the extracts was injected into the analytical column for analysis. The sample flow rate was set at 0.4 mL/min. The MS spectra were acquired in the positive ion mode. The mass fragmentations were identified by using a spectrum database for organic compounds.

### 2.10. Statistical Analysis

The results were expressed as means ± standard deviation of triplicate determinations. Statistical analysis was performed by the IBM Statistical Package for the Social Sciences (SPSS) (version 22) using a two-way analysis of variance (ANOVA) followed by the Tukey multiple comparison post hoc test. Chemical structure and other parameters for each compound were searched using online database software (https://www.chemspider.com and https://www.pubchem.ncbi.nlm.nih.gov). The Xcalibur 2.2 software (Thermo Fisher Scientific, USA) was used for data acquisition and analysis.

## 3. Results and Discussion

### 3.1. Extraction Yield

Secondary metabolites from plants are structurally and chemically diverse; thus, various organic solvents were used as extractants to allow maximal extraction of different types of important bioactive phytochemicals with varying ranges of polarities. It was observed that percentage yields of extraction depended on the polarity indices of the organic solvents. Similar observations were made by Abdisa et al. [55]. Water and methanol extracted had higher percentage yields compared to hexane, dichloromethane (DCM), and acetone. This suggested that the plant material possessed more polar compounds than nonpolar ones.

### 3.2. Antibacterial Activity

The lower the concentration of an extract required to inhibit bacterial growth, the greater the antibacterial activity it possesses. Noteworthy activity was regarded as the concentration of the extracts showing activity at minimum inhibitory concentrations (MIC) less than 1 mg/mL. Some of the antibacterial activity shown by the various extracts was comparable to that of tetracycline (Table 2). Tetracyclines are a class of antibiotics with a broad-spectrum activity against numerous Gram-positive and Gram-negative bacteria by inhibiting protein synthesis [56]. The acetone leaves extracts of *C. bispinosa*, *S. peterisana*, *G. volkensii*, and *C. glabrum* had broad-spectrum antibacterial activities against *S. aureus*, *E. coli*, *P. aeruginosa*, and *M. smegmatis* with MIC values between 0.31–0.63 mg/mL. This broad-spectrum activity of the acetone extracts indicated that the bioactive extracts were nonselective for the type of microbial cell wall. The presence of different types of bioactive phytochemicals in the acetone extracts such as phenolic compounds, the esters of weak acids, fatty acids, terpenes, and others can affect multiple target sites against the bacterial cells [57].

Only the acetone extracts of *G. volkensii* and *C. glabrum* showed inhibitory activity against *M. tuberculosis* at 0.63 and 2.5 mg/mL, respectively. Our results were consistent with previous studies with slight variations where *C. glabrum* was reported to have a MIC of 0.156 mg/mL and 0.312 mg/mL against *M. tuberculosis* and *M. smegmatis*, respectively [58]. Although ethnobotanical studies report *G. volkensii* to be used traditionally for tuberculosis treatment [20], the antimycobacterial activity of this plant has not yet been reported. The antimycobacterial activities reported in this study provide scientific credence to the anti-TB potential of *G. volkensii* leaves. Acetone has been reported to extract various hydrophilic and lipophilic components from plant material [59] and may reflect a higher probability of diffusing through the mycobacterial cell wall.

Despite belonging to the same family, *S. petersiana* and *A. senegal* showed different antibacterial properties against the tested microorganisms. All *S. petersiana* extracts showed significant activity against *S. aureus* while none of *the A. senegal* extracts was active. It has previously been suggested that MIC values of plant extracts can vary due to the differences in the present bioactive compounds and the volatile nature of the compounds [60]. Mudi and Salisu [61] reported that the hexane fraction of *A. senegal* stem bark had good activity against the respiratory tract pathogens such as *Klebsiella pneumoniae* and *Streptococcus pneumoniae.* In this work, the hexane extract of the leaves of *A. senegal* showed the best antibacterial activity against *P. aeruginosa* with an MIC value of 0.16 mg/mL. These results suggest that the leaves may be used as a substitute for the use of stem bark or roots of *A. senegal* to treat bacterial pneumonia. The use of the leaves may contribute to sustainability in the harvest and conservation of *A. senegal*.

One of the important parameters in assessing the microbial activity of plant extracts is total activity (TA). TA (mL) is valuable because it takes into consideration not only the antimicrobial activity but also relates it to the amount of compounds extracted from 1 g of plant material. *G. volkensii* DCM extract had the highest TA against *E. coli* (308 mL), and its acetone extract had a higher TA against *M. smegmatis* (160 mL). The hexane and acetone extracts of *S. petersiana* had the highest TA against *S. aureus* (329 mL) and *P. aeruginosa* (339 mL). The TA of these extracts indicated that their MICs can be diluted to high volumes without disrupting the antibacterial activity. This concept is important when considering further purification or isolation of bioactive compounds from crude extracts [62]. It is known that the active compounds in extracts are commonly produced in lower concentrations, and purification processes further reduce the concentrations of these compounds [63]. Therefore, plant extracts with high TA values serve as good candidates for the isolation and characterisation of bioactive antibacterial compounds. The plant extracts showed bactericidal effects on the tested microorganisms, indicating that the extracts not only inhibit growth but can kill the bacterial cells. The nonselectivity of the plant extracts on the different microorganisms prompted toxicity studies because the antimicrobial activity could be due to toxic compounds in the crude extracts. As such, natural products or their derivatives need to be assessed for their safety before consumption is intended.

### 3.3. Cytotoxicity

Alveolar macrophages are one of the primary phagocytes that are recruited by the host's immune system to clear the air spaces from foreign particles [64]. Vero cells are homologous with human body cells and easy to culture as the use of this cell line has been representative of normal human cells. The sensitivity of the Vero cells to toxicity allows them to be ideal *in vitro* models to be used to evaluate cytotoxicity [65].

**Figure 1:**
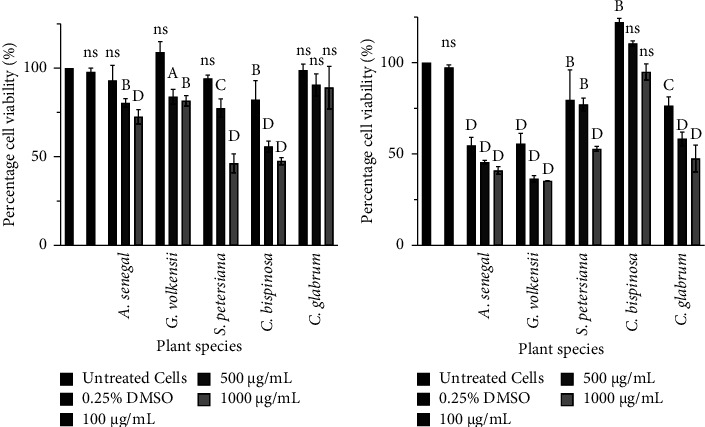
Percentage viability of THP-1 macrophages (a) and vero cell lines (b) after treatment with selected plant extracts. ns: not significant; A: *p* < 0.05; B: *p* < 0.01, C: *p* < 0.001; and D: *p* < 0.0001.

The 0.25% DMSO was used to reconstitute the extracts and was also used as a negative control. The results showed that at the selected concentration of DMSO (0.25%), there was no significant effect on the growth of both the Vero cell line and the THP-1 macrophages (*p* > 0.05). This provided certainty that the solvent did not contribute to the observations. It was notable that each extract exerted different cytotoxic effects on the THP-1 and vero cell lines with varying degrees of significance. At the lowest tested concentration (100 *μ*g/mL), all the extracts did not significantly reduce the cell viability of the THP-1 macrophages (*p* > 0.05); however, *A. senegal*, *G. volkensii*, *S. petersiana*, and *C. glabrum* were toxic to vero cell lines (Figure 1) (*p* < 0.05). *S. petersiana* ethanol extracts were reported to be toxic to vervet monkey cells, exhibiting ID_50_ values of 24 *μ*g/mL [66]. *A. senegal* and *G. volkensii* extracts were generally the most cytotoxic to the vero cell line at all the tested concentrations. The toxicity exerted by the extracts on the normal vero cell lines may be due to challenges the cell lines experience in culture such as being modified by many substances present in the crude extracts [67]. The dichloromethane and methanol extracts of *G. volkensii* twigs/bark have been reported to be toxic by using the micronuclei and comet assays [68]. In addition, the fruit extract (methanol and chloroform) of *G. volkensii* moderately had lethality against brine shrimp (*Artemia Salina*) [22]. Our results were consistent with these previous toxicological studies on *G. volkensii* and demonstrated the cytotoxicity of eukaryotic cells.

*Clerodendrum glabrum* acetone extract was the most nontoxic to THP-1 macrophages with a nonsignificant decrease in viable cells at all tested concentrations. Similarly, *C. glabrum* acetone leaves extracts were previously reported to be nontoxic to the Vero cell line (357.11 *μ*g/mL LC_50_) [58]. The general nontoxicity of *C. glabrum* on different cell lines may explain the lack of toxicity case reports associated with its traditional use. *C. bispinosa* extract was most toxic to the macrophages, suggesting that the immune system may be negatively affected after 24 h exposure. The *C. bispinosa* fruit is popular among locals in KwaZulu-Natal (South Africa) and is commonly sold in the summer season [31]. The widespread use of the fruit and the lack of acute toxicology effects may imply that it is not being consumed in toxic concentrations. It may be worthwhile to further validate the toxicity evaluated in this study by considering *in vivo* experiments where the activity is high enough to justify it. The toxicity of the crude extracts could be due to a toxic compound; thus, the safety/antimicrobial activity can be altered by solvent-solvent fractionation [69].

### 3.4. Anti-Inflammatory Activity

Respiratory tract infections are associated with lung tissue damage where the epithelial cells are the first to be affected. The tissue damage can be both from microbial infection and the inflammatory response of the host. Macrophages produce large amounts of proinflammatory chemical species in response to danger signals [70]. If the injury persists, proinflammatory signals continue and further damage the epithelium of the surrounding cells. For tissue repair to take place and restore normal tissue, inflammation must first be reduced [70].

**Figure 2:**
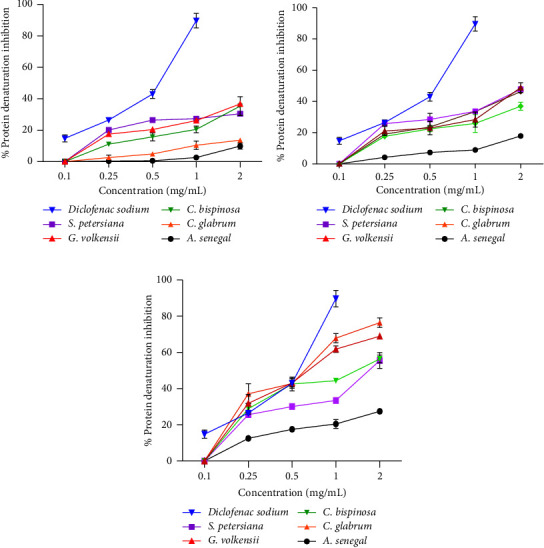
Heat-induced BSA denaturation inhibition by (a) water extracts, (b) methanol extracts, and (c) hexane extracts from the different plant species.

**Figure 3:**
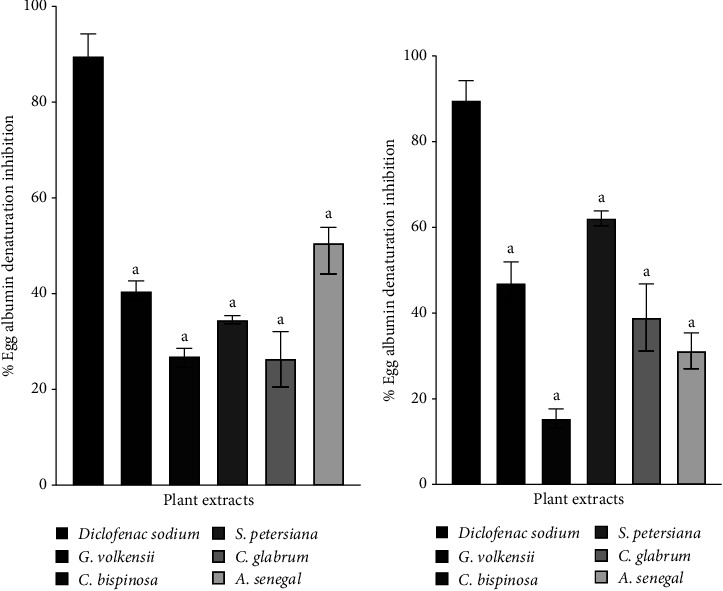
Egg albumin denaturation inhibition of the (a) methanol and (b) water extracts compared to the standard, diclofenac sodium. Significant difference where A: *p* < 0.0001.

Figure 2 represents the bovine serum albumin (BSA) denaturation inhibition of the extracts. As expected, diclofenac sodium showed concentration-dependent*in vitro* BSA denaturation inhibition [45]. Similarly, the plant extracts demonstrated a concentration-dependent inhibition. It was observed that the hexane extracts had better anti-BSA denaturation activity, followed by methanol and water. This suggested that nonpolar compounds had better antiinflammatory activity than polar compounds. At 0.25 and 0.5 mg/mL concentrations, hexane extracts from *C. bispinosa*, *and G. volkensii* demonstrated comparable antiinflammatory activity to diclofenac sodium (Figure 2). *G. volkensii* had the highest anti-BSA denaturation activity among the tested plant species. No notable antiinflammatory studies have previously been reported for *C. bispinosa* and *G. volkensii* leaves extracts. For the former, more research has been conducted into other members of the Carissa genus such as *Carissa caranda* [71] and *Carissa macrocarpa* [72]. The traditional use of *G. volkensii* in the treatment of inflammation-associated diseases and conditions has been reported [20] and may explain the observed anti-inflammatory activities. Only polar solvents, methanol, and water extracts demonstrated antiegg albumin denaturation activity (Figure 3). *S. petersiana* water extract showed good antiegg albumin denaturation activity (>60% inhibition). Similarly, Aremu et al. [73] reported 99.3 ± 1.2% inhibition of cyclooxygenase 1 (COX-1) by *S. petersiana* leaves water extract. The albumin assays demonstrated that the plant extracts have different mechanisms of anti-inflammatory action and may help reduce the negative impact of respiratory diseases which are also characterized by an increase in and prolonged inflammation. In addition, plant extracts with antiprotein denaturation activities have been shown to exhibit thrombolytic and antinociceptive potential in animal models [74, 75].

### 3.5. Antioxidant Activity

DPPH radical scavenging activity and ferric-reducing power expressed as EC_50_ values are presented in Table 3. Low EC_50_ values indicated that a small amount of the extract is required to reduce half of the total amount of the free radical in a solution. The water extracts of *G. volkensii*, *S. petersiana*, and *A. senegal* showed the highest DPPH radical scavenging activity for each plant. In support, water extracts have been reported to possess antioxidant activity because of the high presence of phenolic chemicals [76]. Polyphenolic compounds are associated with antioxidant activity due to their ability to readily donate hydrogen and/or electrons. The highest DPPH radical scavenging activity of all the plant extracts was the *C. bispinosa* hexane extract, indicating that nonpolar phytochemicals in the extract had strong radical scavenging potential (EC_50_ 160.65 ± 0.21 *μ*g/mL). Terpenes have been reported to possess antioxidant activity [77], and this may explain the presence of antioxidant activity in the hexane extract of *C. bispinosa*. Moderate DPPH radical scavenging activity of extracts from *G. volkensii* fruit has previously been reported [22], and flavonoids from the fruit of *C. bispinosa* have been shown to have moderate DPPH radical scavenging activity [32]. Therefore, our antioxidant study suggests that the leaves of *C. bispinosa* and *G. volkensii* are better sources of antioxidant compounds than their fruits. The acetone leaf extract of *G. volkensii* had the greatest ferric-reducing power (123.64 ± 0.41 *μ*g/mL). Ascorbic acid had higher free radical scavenging activity (20.41 ± 0.92 *μ*g/mL) and ferric-reducing power (22.67 ± 1.68 *μ*g/mL) than all the tested extracts. The benefit of the reducing capability of the plant extracts is the reduction of the effects of oxidative stress such as cell and tissue damage which can lead to gradual weakness of the immune system. This may further rationalize the traditional use of these selected plant species for not only the treatment of respiratory tract infections but also the treatment of associated symptoms such as fever, cough, and chest pains.

### 3.6. Phytochemical Analysis

Due to the polarity of phenolics, it was expected that total phenolic content would likely be the highest in the polar solvents. Indeed, phenolic content increased from the hexane, dichloromethane, acetone, methanol, and water extracts of all the plant species (Table 4). The water extracts of *S. petersiana* had the highest total phenolic content (TPC) of 1239.94 ± 0.18 mg GAE/g and total tannin content of 14.14 ± 0.24 mg GAE/g (*p* < 0.05). A flavonoid, luteolin, was previously isolated from the ethanol seed extract of *S. petersiana* [66]. The lowest TPC was detected in the DCM extract of *C. bispinosa* (14.70 ± 0.14 mg GAE/g) followed by *A. senegal* methanolic extract (17.06 ± 0.35 mg GAE/g). The fruit extracts of *C. bispinosa* have also been reported to possess bioactive flavonoids [32]. *A. senegal* water extract possessed significantly higher total proanthocyanidin content (579.54 ± 0.32 mg GAE/g). Similarly, flavonoids, alkaloids, and tannins in the acetone and methanol leaves extracts of *A. senegal* have previously been detected [78]. The hexane and dichloromethane extracts of *G. volkensii* had the lowest proanthocyanidin and tannin content. The results obtained from the investigation of phytochemical content give preliminary data on some important groups that are present in the plants investigated. The phytochemical analysis not only demonstrated the presence of high quantities of bioactive phytochemicals but also showed that the crude extracts indeed consist of a myriad of compounds. Further analysis is required for the identification of specific bioactive compounds to enable a detailed elucidation of the possible mechanisms of action.

### 3.7. Liquid Chromatography-Mass Spectrometry (LC-MS) Studies

LC-MS analysis of the acetone leaves extracts of *S. petersiana* detected *columnidin,* hercynine, L-lysine citrate, gamma-linolenate (GLA) at 5.25, 5.65, 8.35, and 10.48 min retention times, respectively (Table 5). Gamma-linolenate is an essential polyunsaturated fatty acid and the first intermediate in the bioconversion of linolenic acid to the long-chain polyunsaturated fatty acid, arachidonic acid [79]. GLA deficiency has been associated with different physiologic/pathophysiologic conditions [80]. The role of Gamma-linolenate in fatty acid metabolism may explain its presence in *S. petersiana*, *A. senegal*, *G. volkensii*, and *C. bispinosa* and may suggest their nutraceutical potential. Indeed, the nutraceutical potential of the carissa genus has been reported [29]. Cetylpyridinium was detected in *A. senegal* and *C. bispinosa* at 8.47 and 8.50 mins RT. The mechanisms of action of cetylpyridinium have been reported to involve interference with osmoregulation and homeostasis of the bacterial cells, and high concentrations lead to degeneration of the membranes with subsequent leakage of cytoplasmic contents [81].

Previous phytochemical studies of *G. volkensii* leaves reported the presence of various diterpenes and triterpenoids in the crude hexane leaves extracts [82]. In this work, a pentacyclic triterpenoid, cochalate was detected in *G. volkensii* acetone leaves extract with an 11.78 min RT. Consistently, the seed and the pulp of *G. volkensii* have been reported to possess triterpenes [22]. The broad-spectrum antibacterial activities demonstrated by *G. volkensii* leaves extracts may be attributed to the presence of various bioactive terpenes and terpenoids extracted with acetone and hexane. Numerous polyphenolic compounds were detected in the *C. glabrum* acetone extract. In this study, two flavonoids, 7-hydroxy-2-(4-methoxyphenyl)-4-oxo-chroman-5-olate and (3R)-3-(2,4-dimethoxyphenyl)-7-hydroxy-4-oxo-chroman-5-olate were detected at 8.20 and 8.43 mins RT, respectively. Moreover, pentanoic acid (valeric acid) was detected at 5.05 min RT. Valeric acid has been shown to be active against Gram-negative and Gram-positive microorganisms [83]. 3,5-Dimethoxy-benzoic acid was isolated from the leaves of *C. glabrum* cultivated in Egypt [33]. Due to their lipophilic properties, carboxylic acids and carboxylate compounds have been reported to have notable antimycobacterial activity [84]. The presence of these phytochemicals in the *C. glabrum* acetone extract may be contributing to the determined antimycobacterial activity.

## 4. Conclusions

From our research work, we can conclude that the selected medicinal plants demonstrated their potential use as a phytotherapeutic agent toward the treatment of bacterial respiratory tract infections. Notably, the acetone extracts of *G. volkensii* and *C. glabrum* had antimycobacterial activity against *M. tuberculosis* H37Rv. Our results showed that the extracts had anti-inflammatory and antioxidant activities. It is important that the knowledge be documented and the traditional use be given some credence through modern scientific studies. By using this study, the traditional application of the leaves of the plants as remedies toward bacterial infections may be favoured/approved. The isolation of the bioactive compounds and characterisation of the specific mechanisms of action responsible for the observed antibacterial and antiinflammatory activities still require more analysis. Moreover, the use of animal models is required to further validate the anti-TB and antinociceptive potential of the extracts.

## Figures and Tables

**Table 1:** Medicinal plants used in this study.

Plant names (families)	Common names	Parts used	Traditional use	References	Voucher numbers
*Senna petersiana* (bolle) lock (fabaceae)	English: *Dwarf Cassia*, *Eared Cassia*, *Eared Senna*, *Monkey Pod*, *Monkey Senna*, TšhiVenda: *Munembenembe*, uhwabile (isiZulu), sepedi: *Bohlôko*	Root and leaves	Coughs, colds, syphilis, and helminthic infections	[24]	SSS511
*Acacia senegal* (L.) willd (fabaceae)	English: White gum tree	Bark, roots and leaves	Respiratory infections, wounds diseases, inflammation, and stomach aches	[27]	SSS34
*Carissa bispinosa* (L.) desf. Ex brenan (apocynaceae)	English: *Forest num-num*, isiZulu: *isibethankunzi*, *isabetha*	Roots	Cough and diarrhoea	[30]	SSS09
*Clerodendrum glabrum* E. Mey. var. *glabrum* (lamiaceae)	English: Tinderwood, isiZulu: umQoqonga, sepedi: mohlokohloko	Roots and bark	Ulcers, diarrhoea, and coughs	[34]	SSS712
*Gardenia volkensii* (rubiaceae)	English: *Bushveld gardenia*, sepedi: *Morala*, xitsonga: *ntsalala*, isiZulu: *umgongwane*	Fruits and roots	Chest complaints and tuberculosis related infections	[39]	UNIN1220022

**Table 2:** Antibacterial activity of the plants extracts against pathogens.

Plant species	Solvent	*S. aureus*			*E. coli*			*P. aeruginosa*			*M. smegmatis*			*M. tuberculosis*	
		MIC	MBC	TA	MIC	MBC	TA	MIC	MBC	TA	MIC	MBC	TA	MIC	TA
*C. bispinosa*	H	**0.63**	—	65	—	—	—	**0.63**	—	65	2.5	—	59	—	—
	D	**0.63**	—	83	—	—	—	**0.63**	—	83	2.5	—	86	—	—
	A	**0.31**	2.5	151	**0.63**	1.25	74	**0.31**	1.25	151	**0.63**	—	120	—	—
	M	**0.63**	—	311	2.5	—	78	1.25	—	157	—	—	—	—	—
	W	1.25	—	134	2.5	**0.63**	67	2.5	—	67	—	—	—	—	—

*G. volkensii*	H	**0.31**	2.5	239	1.25	—	59	**0.63**	—	118	1.25	—	46	—	—
	D	**0.63**	**0.63**	151	**0.31**	1.25	308	1.25	—	76	2.5	—	34	—	—
	A	**0.31**	**0.63**	293	**0.31**	1.25	294	**0.63**	1.25	145	**0.63**	—	160	2.5	45
	M	**0.63**	—	250	1.25	—	126	2.5	—	63	2.5	—	21	—	—
	W	2.5	2.5	74	—	2.5	—	—	—	—	—	—	—	—	—

*S. petersiana*	H	**0.31**	**0.63**	329	**0.63**	—	162	**0.63**	—	162	2.5	—	14	—	—
	D	**0.63**	2.5	161	1.25	—	81	1.25	—	81	—	—	—	—	—
	A	**0.63**	—	167	1.25	**0.63**	167	**0.31**	**0.63**	339	**0.63**	—	118	—	—
	M	**0.31**	—	361	1.25	1.25	89	1.25	—	89	—	—	—	—	—
	W	**0.16**	2.5	773	—	**0.63**	—	**0.63**	—	196	2.5	2.5	35	—	—

*A. senegal*	H	—	—	—	2.5	**0.63**	64	**0.16**	**0.63**	89	—	—	—	—	—
	D	—	—	—	**0.63**	**0.31**	46	**0.63**	1.25	46	—	—	—	—	—
	A	—	—	—	**0.31**	**0.31**	81	**0.63**	**0.63**	40	—	—	16	—	—
	M	—	—	—	2.5	2.5	44	2.5	—	44	—	—	—	—	—
	W	—	—	—	2.5	—	102	—	—	—	—	—	—	—	—

*C. glabrum*	H	**0.31**	**0.63**	55	1.25	1.25	87	**0.31**	—	121	—	—		—	—
	D	**0.63**	—	82	—	1.25	94	**0.63**	**0.31**	293	—	—		—	—
	A	1.25	—	134	1.25	**0.31**	120	**0.31**	1.25	250	**0.63**	—	115	**0.63**	115
	M	**0.63**	—	239	1.25	**0.63**	110	**0.63**	2.5	74	2.5	—	42	—	—
	W	—	—	—	—	—	—	—	—	—	—	—	—	—	—

Antibiotic														—	—

Tetracycline		**0.21**			**0.13**			**0.16**						—	—

Rifampicin											**0.0016**			**0.002**	

MIC, minimum inhibitory concentration (mg/mL); MBC, minimum bactericidal concentration (mg/mL); TA, total activity (mL); H, hexane; D, dichloromethane; A, acetone; M, methanol; and W, water. Notable antibacterial activity (MIC and MBC values < 1 mg/mL) is bolded, (—): undetected activity at highest tested concentration (2.5 mg/mL).

**Table 3:** Antioxidant activity of plant extracts expressed as EC_50_.

Plant species	Solvent	Free-radicalscavenging activity	Ferric-reducing power
		EC_50_ (*μ*g/mL)^*∗*^	
*C. bispinosa*	H	160.65 ± 0.21^a^	237.8 ± 0.10^i^
	D	514.57 ± 0.28^i^	411.03 ± 0.15^k^
	A	312.83 ± 0.16^d^	308.82 ± 0.13^j^
	M	452.06 ± 0.42^h^	3688.67 ± 3.31^s^
	W	205.78 ± 0.46^b^	139.92 ± 0.11^b^

*G. volkensii*	H	559.95 ± 0.37^k^	219.3 ± 0.22^h^
	D	568.32 ± 0.99^l^	213.59 ± 0.35^g^
	A	442.8 ± 0.46^g^	123.64 ± 0.41^a^
	M	376.66 ± 0.81^f^	210.47 ± 0.23^g^
	W	543.85 ± 0.78^j^	1135 ± 0.82^o^

*S. petersiana*	H	718.85 ± 0.59^p^	1223.58 ± 1.03^p^
	D	889.76 ± 0.77^r^	1231.25 ± 0.35^q^
	A	636.56 ± 0.77^m^	1127.08 ± 1.03^n^
	M	670.12 ± 0.5^o^	1558.33 ± 0.27^r^
	W	271.77 ± 0.76^c^	178.23 ± 0.107^d^

*A. senegal*	H	353.80 ± 0.07^e^	193.05 ± 0.10^f^
	D	864.30 ± 0.42^q^	188.64 ± 0.21^e^
	A	646.42 ± 0.35^n^	158.33 ± 0.19^c^
	M	932.40 ± 0.32^s^	726.83 ± 0.12^m^
	W	272.56 ± 0.21^c^	493.28 ± 1.72^l^

*C. glabrum*	H	170.88 ± 0.19^a,b^	229.71 ± 0.61^h,i^
	D	353.53 ± 0.21^e^	1106 ± 2.86^n^
	A	160.58 ± 0.54^a^	139.62 ± 0.23^b^
	M	326.55 ± 0.34^e,f^	207.95 ± 0.25^f,g^
	W	536.46 ± 0.23^j^	1455.17 ± 3.41^r^

Ascorbic acid		20.41 ± 0.92	22.67 ± 1.68

EC_50_, half maximal effective concentration; H, hexane; D, dichloromethane; A, acetone; M, methanol; W, water. ^*∗*^Values expressed as mean ± standard deviation (SD) of triplicate experiments; values with different letter superscripts in a column are significantly different at *p* < 0.05; same letter superscript values in a column are not significantly different (*p* > 0.05).

**Table 4:** Phytochemical contents of the different plant extracts.

Plant species	Solvent	Total phenolic content (mg·GAE/g)^*∗*^	Total tannin content (mg·GAE/g)^*∗*^	Total proanthocyanidins content (mg·GAE/g)^*∗*^	Total flavonoid content (mg·QE/g)^*∗*^	Total flavonols content (mg·QE/g)^*∗*^	Total alkaloid content (mg·APE/g)^*∗*^
*C. bispinosa*	H	14.70 ± 0.14^a^	13.06 ± 0.03^n^	183.06 ± 0.11^f^	6.79 ± 0.17^h^	64.77 ± 0.04^p^	13.38 ± 0.28^c,d^
	D	35.80 ± 0.30^d^	9.91 ± 0.03^k^	97.17 ± 0.30^d^	10.36 ± 0.05^i^	57.20 ± 0.07^n^	10.62 ± 0.17^b^
	A	92.27 ± 0.25^g^	10.39 ± 0.07^k^	241.59 ± 0.05^g^	7.51 ± 0.04^h^	77.59 ± 0.11^r^	11.72 ± 0.11^b,c^
	M	106.73 ± 0.25^h^	6.39 ± 0.15^h,i^	106.74 ± 0.32^e^	16.18 ± 0.06^k^	16.66 ± 0.06^i^	13.26 ± 0.05^b,c,d^
	W	137.52 ± 0.07^i^	12.79 ± 0.02^n^	13.22 ± 0.29^b^	4.98 ± 0.10^f,g^	5.41 ± 0.04^d^	217.48 ± 0.93^h^

*G. volkensii*	H	172.07 ± 0.14^j^	1.01 ± 0.01^a^	0.18 ± 0.43^a^	1.52 ± 0.27^a,b^	1.25 ± 0.71^b^	1.54 ± 0.44^a^
	D	277.01 ± 0.07^l^	1.49 ± 0.03^a,b^	0.07 ± 0.05^a^	5.86 ± 0.87^e,f,g^	0.087 ± 0.43^a^	874.30 ± 0.67^m^
	A	440.06 ± 0.34^m^	5.87 ± 0.04^g,h^	44.95 ± 0.03^c^	4.26 ± 0.85^d,e,f^	2.76 ± 0.32^c^	78.26 ± 0.29^f^
	M	582.78 ± 0.25^n^	7.36 ± 0.05^j^	1.39 ± 0.39^a^	6.11 ± 0.61^g,h^	5.06 ± 0.01^e^	16.36 ± 1.45^e^
	W	1000.43 ± 0.12^s^	12.14 ± 0.6^m^	266.73 ± 0.61^h^	9.56 ± 0.01^i^	23.09 ± 0.10^k^	370.09 ± 0.67^k^

*S. petersiana*	H	661.22 ± 0.07^o^	11.43 ± 0.56^l^	398.49 ± 0.52^l^	2.30 ± 0.20^a,b,c^	6.41 ± 0.16^f^	10.63 ± 0.01^b^
	D	722.94 ± 0.18^p^	4.31 ± 0.10^e,f^	506.33 ± 0.81^o^	3.52 ± 0.34^c,d,e,f^	7.17 ± 0.383^g^	0.38 ± 0.78^a^
	A	787.90 ± 0.07^q^	5.62 ± 0.04^g^	340.66 ± 0.74^j^	4.05 ± 0.54^d,e,f^	13.32 ± 0.15^h^	0.03 ± 0.63^a^
	M	845.81 ± 0.07^r^	3.81 ± 0.03^d,e^	576.91 ± 0.69^p^	3.01 ± 0.29^b,c,d^	0.08 ± 0.04^a^	14.91 ± 0.51^d,e^
	W	1239.04 ± 0.14^t^	14.14 ± 0.24^o^	416.66 ± 0.62^m^	3.24 ± 0.25^c,d,e^	0.09 ± 0.01^a^	15.76 ± 0.32^d,e^

*A. senegal*	H	17.06 ± 0.35^b^	6.86 ± 0.02^i,j^	579.54 ± 0.32^q^	13.42 ± 0.65^j^	62.10 ± 0.06^o^	100.68 ± 1.54^g^
	D	23.03 ± 0.18^c^	4.92 ± 0.02^f^	356.76 ± 0.08^k^	7.18 ± 0.05^h^	41.55 ± 0.16^m^	354.92 ± 1.68^j^
	A	49.29 ± 0.12^e^	2.93 ± 0.04^c^	505.25 ± 0.29^o^	10.86 ± 0.45^i^	67.58 ± 0.06^q^	244.99 ± 0.34^i^
	M	57.87 ± 0.068^f^	1.81 ± 0.04^b^	441.86 ± 0.23^n^	6.69 ± 0.04^h^	28.60 ± 0.03^l^	788.69 ± 0.77^l^
	W	250.02 ± 0.18^k^	3.49 ± 0.02^c,d^	297.07 ± 0.32^i^	1.19 ± 0.04^a^	17.64 ± 0.10^j^	14.78 ± 0.73^d,e^

*C. glabrum*	H	94.01 ± 0.18^g^	9.61 ± 0.01^k^	296.82 ± 0.08^i^	15.93 ± 0.47^k^	63.76 ± 0.06^p^	100.72 ± 0.34^g^
	D	24.96 ± 0.18^c^	7.32 ± 0.03^j^	573.11 ± 0.27^p^	19.75 ± 0.41^l^	130.43 ± 0.15^s^	993.48 ± 1.60^n^
	A	65.14 ± 0.07^f,g^	12.96 ± 0.01^m^	579.11 ± 0.03^q^	27.84 ± 0.29^m^	129.85 ± 0.06^s^	727.02 ± 1.46^l,m^
	M	107.40 ± 0.12^h^	3.49 ± 0.01^c,d^	297.69 ± 0.05^i^	7.63 ± 0.04^h^	41.01 ± 0.14^m^	100.97 ± 0.17^g^
	W	35.95 ± 0.14^d^	1.63 ± 0.02^b^	124.5 ± 0.30^e,f^	1.18 ± 0.04^a^	0.08 ± 0.001^a^	323.02 ± 0.50^j^

H, hexane; D, dichloromethane; A, acetone; M, methanol; W, water; mg GAE/g, milligram of gallic acid equivalence/gram of extract; mg QE/g, milligram quercetin equivalence/gram of extract; mg AE/g, milligram atropine equivalent/gram of extract. ^*∗*^Values expressed as the mean ± standard deviation (SD) of triplicate experiments; values with different letter superscripts in a column are significantly different at *p* < 0.05; same letter superscript values in a column are not significantly different (*p* > 0.05).

**Table 5:** Liquid chromatography-mass spectrometry analysis of acetone crude extracts.

Retention time (mins)	Ions	IUPAC names (common names)	Formulae	Mono isotopic masses
*S. petersiana*				
4.95	M^+^H^+^	3,3′,4′,5,5′,7-hexahydroxyflavylium	C_15_H_11_O_7_	303.051
5.25	M^+^H^+^	(2S)-2-(3,4-dihydroxyphenyl)-7-hydroxy-4-oxo-chroman-5-olate (columnidin)	C_15_H_11_O_6_	287.056
5.65	M^+^H^+^	(2S)-3-(1H-imidazole-5-yl)-2-(trimethylazaniumyl)propanoate (hercynine)	C_9_H_15_N_3_O_2_	197.117
6.55	M^+^H^+^	4-ethyl-3-methyl-1-(octoxymethyl)pyridin-1-ium	C_17_H_30_NO	264.233
8.35	M^+^H^+^	(2S)-2,6-diaminohexanoic acid; 2-hydroxypropane-1,2,3-tricarboxylic acid (L-Lysine citrate)	C_12_H_22_N_2_O_9_	338.132
8.99	M^+^H^+^	4-[[2-(4-ethoxycarbonyl-3,5-dimethylpyrazol-1-yl)pyridin-3-yl]amino]-4-oxobutanoate	C_17_H_19_N_4_O_5_	359.136
10.48	M^+^H^+^	(6Z,9Z,12Z)-octadeca-6,9,12-trienoate (gamma-linolenate)	C_18_H_29_O_2_^−^	277.217
12.15	M^+^H^+^	N-ethyl-N-oxidotetradecan-1-amine	C_16_H_34_NO^−^	256.264
*A. senegal*				

1.25	M^+^H^+^	2,3,4-tris[(2-aminoethylamino)methyl]phenol	C_15_H_30_N_6_O	310.249
3.59	M^+^H^+^	Methyl (Z,12R)-12-hydroxyoctadec-9-enoate (methyl ricinoleate)	C_19_H_36_O_3_	312.266
5.03	M^+^H^+^	2-[[(2R)-1-[[(1S)-2-[(4-carbamimidoylphenyl)methylamino]-2-oxo-1-piperidin-4-ylethyl]amino]-3-cyclohexyl-1-oxopropan-2-yl]amino]acetic acid	C_26_H_40_N_6_O_4_	500.312
5.65	M^+^H^+^	(1S,2R,3S,4S)-3-hydroxy-4,7,7-trimethylbicyclo[2.2.1]heptane-2-carboxylate	C_11_H_17_O_3_^−^	197.118
6.95	M^+^H^+^	2-cyanoethyl-(3-phenoxyphenyl)azanide	C_15_H_13_N_2_O^−^	237.103
7.76	M^+^H^+^	(E)-3-cycloheptyl-2-methylprop-2-enoate	C_11_H_17_O_2_^−^	181.124
8.45	M^+^H^+^	1-hexadecylpyridin-1-ium (cetylpyridinium)	C_21_H_38_N^+^	304.301
9.56	M^+^H^+^	(6Z,9Z,12Z)-octadeca-6,9,12-trienoate (gamma-linolenate)	C_18_H_29_O_2_^−^	277.217
10.83	M^+^H^+^	3-methoxy-5,6,7,8-tetrahydronaphthalene-2-carboxylate	C_12_H_13_O_3_^−^	205.086
12.30	M^+^H^+^	N-(3-methoxypropyl)-1-[1-methyl-3-[(E)-[3-oxo-5-(3-pyridylcarbamoylamino)benzofuran-2-ylidene]methyl]pyrrolo[2,3-b]pyridin-4-yl]piperidine-4-carboxamide	C_33_H_35_N_7_O_5_	609.270
12.95	M^+^H^+^	(4R)-5-[3-(4,4-diphenyl-1-piperidyl)propylcarbamoyl]-2,6-dimethyl-4-(4-nitrophenyl)-1,4-dihydropyridine-3-carboxylate	C_35_H_37_N_4_O_5_	593.275
*Gardenia volkensii*				

5.68	M^+^H^+^	(1S,2R,3S,4S)-3-hydroxy-4,7,7-trimethylbicyclo[2.2.1]heptane-2-carboxylate	C_11_H_17_O_3_^−^	197.118
7.44	M^+^H^+^	2-(7,8-diacetoxy-4-methyl-2-oxo-chromen-3-yl)butanoate	C_18_H_17_O_8_	361.092
9.67	M^+^H^+^	(6Z,9Z,12Z)-octadeca-6,9,12-trienoate (gamma-linolenate)	C_18_H_29_O_2_^−^	277.217
11.78	M^+^H^+^	(4aR,5S,6aR,6aS,6bR,8aR,10S,12aR,14bS)-5,10-dihydroxy-2,2,6a,6b,9,9,12a-heptamethyl-1,3,4,5,6,6a,7,8,8a,10,11,12,13,14b-tetradecahydropicene-4a-carboxylate (Cochalate)	C_30_H_47_O_4_^−^	471.347
12.34	M^+^H^+^	N-(3-methoxypropyl)-1-[1-methyl-3-[(E)-[3-oxo-5-(3-pyridylcarbamoylamino)benzofuran-2-ylidene]methyl]pyrrolo[2,3-b]pyridin-4-yl]piperidine-4-carboxamide	C_33_H_35_N_7_O_5_	609.27
12.75	M^+^H^+^	(4R)-5-[3-(4,4-diphenyl-1-piperidyl)propylcarbamoyl]-2,6-dimethyl-4-(4-nitrophenyl)-1,4-dihydropyridine-3-carboxylate	C_35_H_37_N_4_O_5_	593.276
*C. bispinosa*				

4.95	M^+^H^+^	5-[(3Z)-3-(3-methyl-2H-isoxazol-5-ylidene)isoxazol-5-yl]-3-nitro-1,2-benzoquinone	C_13_H_9_N_3_O_6_	303.049
5.25	M^+^H^+^	2-(4-pentylcyclohexyl)acetate	C_13_H_23_O_2_^−^	211.170
5.65	M^+^H^+^	(1S,2R,3S,4S)-3-hydroxy-4,7,7-trimethylbicyclo[2.2.1]heptane-2-carboxylate	C_11_H_17_O_3_^−^	197.118
6.54	M^+^H^+^	4-ethyl-3-methyl-1-(octoxymethyl)pyridin-1-ium	C_17_H_30_NO	264.233
7.80	M^+^H^+^	(E)-3-cycloheptyl-2-methylprop-2-enoate	C_11_H_17_O_2_^−^	181.123
8.50	M^+^H^+^	1-hexadecylpyridin-1-ium (Cetylpyridinium)	C_21_H_38_N^+^	304.300
8.78	M^+^H^+^	4-[2-[[(2S)-butan-2-yl]amino]-2-oxoethyl]-N-(2-tert-butylpyrimidin-5-yl)piperazine-1-carboxamide	C_19_H_32_N_6_O_2_	376.259
9.30	M^+^H^+^	(4-octoxyphenyl)azanide	C_14_H_22_NO^−^	220.170
9.69	M^+^H^+^	(6Z,9Z,12Z)-octadeca-6,9,12-trienoate (gamma-linolenate)	C_18_H_29_O_2_^−^	277.217
10.89	M^+^H^+^	7-methoxytetralin-6-carboxylate	C_12_H_13_O_3_	205.087
11.64	M^+^H^+^	N,N-diethyl-4-methylpiperazine-1-carboxamide (diethylcarbamazine)	C_10_H_21_N_3_O	199.169
12.05	M^+^H^+^	(4aS,6aR,6aS,6bR,8aR,10S,12aR,14bS)-10-hydroxy-2,2,6a,6b,9,9,12a-heptamethyl-1,3,4,5,6,6a,7,8,8a,10,11,12,13,14b-tetradecahydropicene-4a-carboxylate (oleanolate)	C_30_H_47_O_3_^−^	455.353
12.15	M^+^H^+^	N-ethyl-N-oxidotetradecan-1-amine	C_16_H_34_NO^−^	256.265
12.74	M^+^H^+^	(4R)-5-[3-(4,4-diphenyl-1-piperidyl)propylcarbamoyl]-2,6-dimethyl-4-(4-nitrophenyl)-1,4-dihydropyridine-3-carboxylate	C_35_H_37_N_4_O_5_	593.276
*C. glabrum*				

3.95	M^+^H^+^	1-chroman-6-yl-5,7-dihydroxy-2,2-dimethyl-nonan-1-one	C_20_H_30_O_4_	334.213
5.05	M^+^H^+^	(2S)-2-[[4-[(2-amino-4-oxo-1H-pteridin-6 yl)methylamino]benzoyl]amino]-5-[3-[[(2R)-2,4-dihydroxy-3,3-dimethyl-butanoyl]amino]propanoyloxy]-5-oxo-pentanoic acid	C_28_H_34_N_8_O_10_	642.239
7.55	M^+^H^+^	N-[(1S)-5-amino-1-[[(1S)-5-amino-1-[[(1S)-1-formyl-4-guanidino-butyl]carbamoyl]pentyl]carbamoyl]pentyl]-4-oxo-chromene-2-carboxamide	C_28_H_42_N_8_O_6_	586.322
7.75	M^+^H^+^	Tert-butyl (1S,2R,3R)-1″-benzyl-3-benzyloxy-2″-oxo-2-(3-phenylpropanoylamino)spiro[cyclohexane-1,3″-indoline]-5″-carboxylate	C_41_H_44_N_2_O_5_	644.325
8.20	M^+^H^+^	7-hydroxy-2-(4-methoxyphenyl)-4-oxo-chroman-5-olate	C_16_H_13_O_5_	285.077
8.43	M^+^H^+^	(3R)-3-(2,4-dimethoxyphenyl)-7-hydroxy-4-oxo-chroman-5-olate	C_17_H_15_O_6_	315.087
10.85	M^+^H^+^	2-(1,1-dimethyl-2-oxo-ethyl)-3-methyl-benzoate	C_12_H_13_O_3_	205.087
12.33	M^+^H^+^	(2R,4R,4aS,5R,5aS,6R,11aS,12aR)-3,5,10,12,12a-pentahydroxy-4-isopropyl-4a,5a,6-trimethyl-1,11-dioxo-9-(phenylcarbamoylamino)-3,4,5,6,11a,12-hexahydro-2H-tetracene-2-carboxamide	C_32_H_39_N_3_O_9_	609.269

## Data Availability

The data used to support the findings of this study are available from the corresponding authors upon reasonable request.
